# Real-Time Gait Event Detection Based on Kinematic Data Coupled to a Biomechanical Model †

**DOI:** 10.3390/s17040671

**Published:** 2017-03-24

**Authors:** Stefan Lambrecht, Anna Harutyunyan, Kevin Tanghe, Maarten Afschrift, Joris De Schutter, Ilse Jonkers

**Affiliations:** 1 Division PMA, Department of Mechanical Engineering, Katholieke Universiteit Leuven, 3000 Leuven, Belgium; stefan.lambrecht@kuleuven.be (S.L.); kevin.tanghe@kuleuven.be (K.T.); joris.deschutter@kuleuven.be (J.D.S.); 2 Department of Biomedical Kinesiology, Katholieke Universiteit Leuven, 3000 Leuven, Belgium; Maarten.afschrift@kuleuven.be; 3 AI Laboratory, Vrije Universiteit Brussel, 1050 Ixelles, Belgium; anna.harutyunyan@vub.ac.be

**Keywords:** gait segmentation, modeling, real-time event detection, adaptive thresholds, neuroprostheses, neurorobotics, kinematics

## Abstract

Real-time detection of multiple stance events, more specifically initial contact (IC), foot flat (FF), heel off (HO), and toe off (TO), could greatly benefit neurorobotic (NR) and neuroprosthetic (NP) control. Three real-time threshold-based algorithms have been developed, detecting the aforementioned events based on kinematic data in combination with a biomechanical model. Data from seven subjects walking at three speeds on an instrumented treadmill were used to validate the presented algorithms, accumulating to a total of 558 steps. The reference for the gait events was obtained using marker and force plate data. All algorithms had excellent precision and no false positives were observed. Timing delays of the presented algorithms were similar to current state-of-the-art algorithms for the detection of IC and TO, whereas smaller delays were achieved for the detection of FF. Our results indicate that, based on their high precision and low delays, these algorithms can be used for the control of an NR/NP, with the exception of the HO event. Kinematic data is used in most NR/NP control schemes and is thus available at no additional cost, resulting in a minimal computational burden. The presented methods can also be applied for screening pathological gait or gait analysis in general in/outside of the laboratory.

## 1. Introduction

Walking neuro-robotics (NR) and -prosthetics (NP) are currently available as therapeutic tools in rehabilitation [1,2], or as permanent assistive devices [3]. Most recent NR and NP, however, do not yet incorporate effective strategies to dynamically interface with the human body using the natural dynamics of the gait process. Synthesizing the walking behaviour in real time would enable the NR/NP to assist and provide augmented proprioceptive inputs synchronized with the task execution. One way to synthesize gait is through the identification of gait and stance events.

In gait laboratories, force platforms are typically used to detect initial contact (IC) and toe off (TO), thus separating gait cycles as well as separating stance from swing phase within one gait cycle. In combination with marker data, additional stance events such as foot flat (FF) and heel off (HO) can also be detected automatically, albeit offline [4]. In wearable applications, these external reference systems cannot be used, and often real-time processing is desired or needed. Pattern recognition approaches, in combination with inertial sensors, have therefore become popular to identify IC and TO [5,6,7]. Current algorithms predominantly use one single sensor attached to each shank. TO and/or IC are typically detected as the minimum shank angular velocity that respectively precedes and follows the maximum velocity, coinciding with mid-swing (MS) [5,6,8]. However, the relation between TO and this minimum in shank angular velocity has since long been debated [5,8,9]. Botzel et al. therefore recently proposed a new TO definition, suggesting that TO corresponds to the midpoint between the minimum shank angular velocity and its zero-crossing [7].

In routine gait analysis, timing of IC and TO is imperative and often sufficient; whereas in ankle-foot orthoses (AFO), control for drop foot patients, and accurate and timely HO detection is fundamental to avoid falls due to stumbling [3,10]. Automatic real-time detection of additional stance events, such as FF and HO would allow these events to be incorporated in the control of NR/NP [3,4,10,11]. The benefits of a more fine-grained segmentation in NR/NP control were already demonstrated in [3]. For the variable-impedance control of an AFO, Blaya and Herr separated the gait cycle into two stance phases and one swing phase. This allowed reduction of foot slapping following IC and enabled higher powered plantar-flexion, resulting in a gait pattern more similar to healthy gaits for subjects with drop foot [3]. They segmented gait cycles based on information from force sensitive resistors (FSRs). Pappas et al. also used a gyroscope on each foot in combination with FSRs to detect IC, FF, HO and TO with the intention to implement this in an NP. They validated their algorithm both indoors and outdoors on healthy subjects and on subjects with impaired gait [12]. FSRs, however, have strong limitations regarding mechanical wear and reliability, and are therefore best avoided in practical applications [13,14].

Kotiadis et al. presented inertial gait phase detection algorithms to trigger drop foot stimulators without the need for FSRs. Four algorithms identifying IC and HO, based on a combination of gyroscopes and accelerometers, were validated offline on a single subject. The presented methods were threshold based, in order to avoid high computational costs. The thresholds were optimized and maintained constant for the one subject tested, and FF or TO were not detected [11]. Recently, Chia et al. [6] presented a threshold-based real-time algorithm where the thresholds were automatically calibrated to the subject and updated at each step. This algorithm was validated on both healthy subjects and stroke patients, but only detected IC and TO [6].

Traditionally, a balance has been sought between minimal instrumentation and reliable and accurate event detection. However, most NR/NP control strategies require continuous real-time monitoring of the kinematic data of all actuated joints. This data could thus also be used to identify gait and stance events at no additional cost. Joint kinematics in NR/NP are typically obtained either through inertial sensors, as is the case in wearable motion analysis applications such as Outwalk [15], or through encoders or potentiometers embedded in the NR. Furthermore, with exception from the method presented in Chia [6], all methods either relied on offline training, offline processing, or at best ran in quasi-real-time with an inherent delay due to signal processing and/or event definition. This despite the established knowledge that optimal delays for control should not exceed 100–125 ms [16], with 61 ms being the smallest delay noticeable by subjects [17].

The objective of our work was to identify the different stance phases in real time with a mean delay below 61 ms and variability smaller than 125 ms from the real event to enable their use in NR/NP control algorithms. In this paper, we therefore present a workflow that enables detection of four gait events (IC, FF, HO, TO) in real time, based on kinematic data commonly available in NR/NP. No sensors other than those already present for the control of the NP/NR are required, and cost and reliability of the NP/NR thus remain unchanged. We developed three kinematic-based real-time gait and stance phase detection algorithms (RTGSD), all using this workflow. Kinematic data and joint angles were coupled to a biomechanical model from which the signals used for event detection by the respective algorithms were extracted. In this paper, we used optical marker data and obtained the joint kinematics offline. Nevertheless, any system providing kinematics off/online can be used as a substitute to be coupled to the biomechanical model and RTGSD-algorithm. Performance of the developed algorithms was evaluated on timing and precision, using an offline dataset of healthy subjects walking on a treadmill.

## 2. Experimental Section

### 2.1. Data Collection and Processing

**Figure 1:**
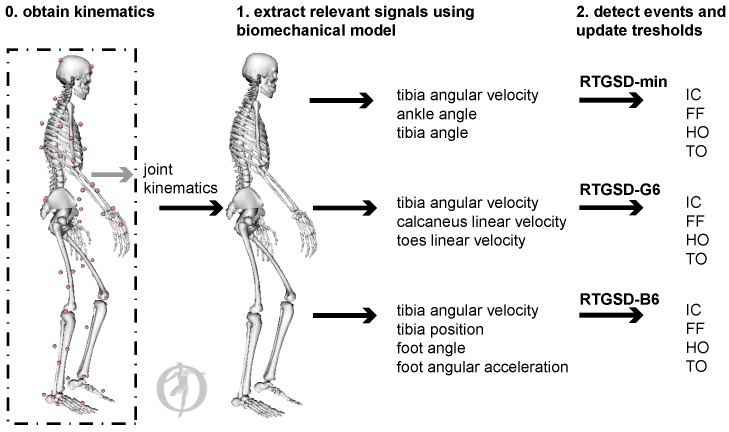
Musculoskeletal model and data processing. The generic musculoskeletal model (3DGaitModel2392) was scaled to the subject’s dimensions. The plug-in gait marker placement protocol was used for the data collection. Joint kinematics were obtained from the measured marker data using a Kalman Smoothing algorithm [20]. In our study, joint kinematics were obtained offline (discontinuous box), but this can be substituted by any online method to obtain joint kinematics. The real-time processing, outside of the discontinuous box, consisted of feeding the joint kinematics back to the scaled model, extracting the information required by the respective algorithms from this model and performing the gait and stance phase detection.

Seven healthy subjects (61 ± 8 kg, 23 ± 3 years) signed a written informed consent to participate in this study; the ethical committee of UZ Leuven gave approval to the experimental protocol. Each subject performed three walking trials on an instrumented split-belt treadmill at, respectively, 3, 4, and 5 km/h. In total, 558 steady state steps were analysed. A motion capture system consisting of 10 infrared cameras recorded the motion of reflective markers attached to anatomical locations of the subject, according to the extended plug in gait marker protocol (100 Hz) (Figure 1). The generic “3DGaitModel2392” musculoskeletal model was scaled to the subject’s dimensions using Opensim 3.3 [18,19]. A Kalman smoothing algorithm was then used to compute the joint kinematics of the scaled model that best reproduced the measured motion of the markers [20]. These steps are represented by the discontinuous box in Figure 1 and can be substituted by real-time kinematics obtained by the NP/NR. The obtained joint kinematics were then fed-back to the scaled model to extract the data required by each of the algorithms (Figure 1). Since real-time kinematics were not available from marker data, all processing was done offline for this study. However, when real-time kinematics are available, the entire process can be performed online (Figure 1). In [21], a previous version of RTGSD-min was implemented on a BeagleBone Black (BeagleBoard, Oakland, MI, USA) and ran in real time. In ongoing pilot work, we implemented RTGSD-G6, using exoskeleton encoder data as input, in real time using the entire workflow as presented here. However, no independent and reliable reference data for the stance events were available for those trials. AMTI force plates embedded in the split-belt treadmill measured the ground reaction forces at 1000 Hz.

All processing and validation was performed in Matlab (2014b, The Mathworks, Natick, MA, USA). The events as identified by the proposed algorithms were compared to the reference data obtained from the force platforms embedded in the treadmill (IC, TO) and marker data (FF, HO). The precision of the algorithms was quantified by contrasting the True Positives (TP) against the sum of the TP and the False Positives (FP). False Negatives (FN) were also reported. Timing was assessed using the Bland–Altman method [22].

### 2.2. Reference Events

**Figure 2:**
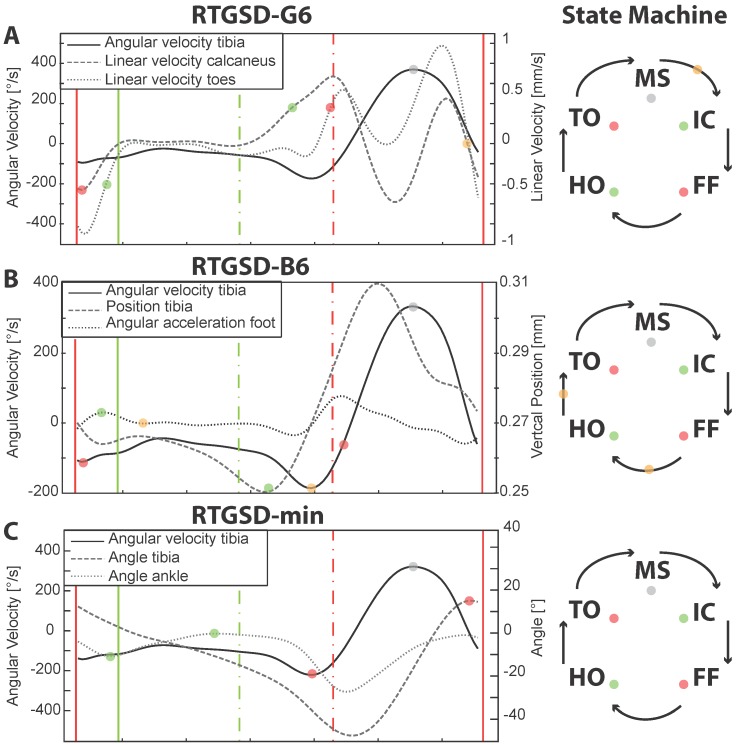

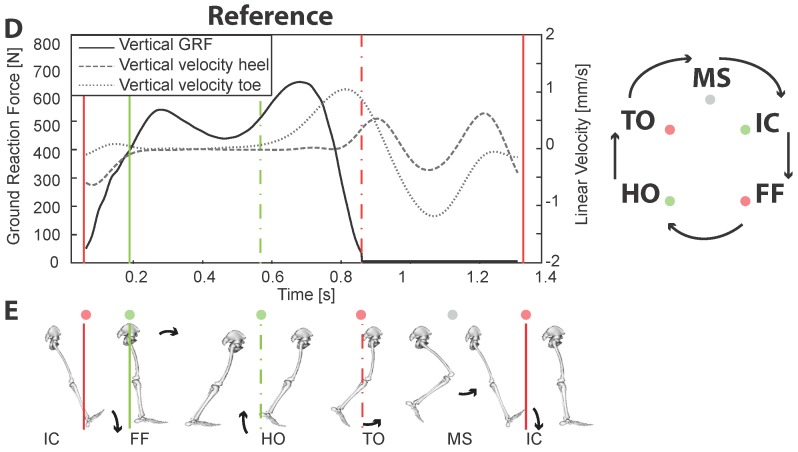
Gait and stance events detected by each of the presented algorithms. All events for one step are represented in (**E**) (IC, FF, HO, TO, MS). In (**A**–**D**) the black lines are plotted against the axis on the left, whereas the grey lines are plotted against the axis on the right. A single right step from one of the subjects is shown. (**D**) displays the reference events. (**C**) displays the events as detected for this step by the RTGSD-min algorithm. In (**B**), the events as detected by RTGSD-B6 are represented. The events as detected by RTGSD-G6 are shown in (**A**). The orange dots in (**A**,**B**) represent flags that are used by the respective state-machines to detect the next event. In (**A**), the zero-crossing towards a minimum of the calcaneus vertical velocity is flagged, enabling subsequent detection of IC. In (**B**), the zero crossing of the foot acceleration and the minimum of shank angular velocity are flagged, enabling the subsequent detection of HO and TO.

Reference events, more specificically, IC, TO, FF and HO, were defined based on the force plate and marker data (Figure 2, panel D). IC and TO were identified using a 20 N threshold on the vertical force component measured by the force plates (Figure 2, panel D solid line). FF and HO were defined according to the algorithm presented in [4], using a 100 mm/s threshold on, respectively, the toe (dotted line) and heel (dashed line) marker vertical velocities (Figure 2, panel D). The original method was developed for over-ground gait using sagittal plane marker velocity. To accommodate to the treadmill data, only the vertical velocity was used in this study. We validated this modification by comparing the timing of the IC and TO events using only vertical velocity thresholds to the GRF-based (Ground Reaction Forces) reference events. Mean delays of −43 ms for TO and −3 ms for IC were observed when compared to the force plate data. These results are similar or better than those presented in [4]. Therefore, the modified method using only the vertical marker velocity was assumed to correctly identify FF and HO.

### 2.3. RTGSD Structure

The developed real-time gait and stance phase detection (RTGSD)-algorithms share a double-layered structure based on state-machines for the real-time detection (Algorithm 1) and a parallel layer updating the thresholds (Algorithm 2).

In the real-time detection layer, events are detected based on the real-time signals extracted from the model, the current state of the state machine, and the current thresholds. To detect an event, the state-machine of the leg of interest has to be in the correct state, and the amplitude of the target signal for that event had to exceed the corresponding threshold (Figure 2) (Algorithm 1). The signals used by each of the algorithms are listed in Figure 1. In Figure 2, these signals are plotted in the respective panels, and the events are marked by coloured circles both in the plot and in the state-machine. The event definitions used by each of the algorithms are also listed in Table 1. The state-machines of each leg operated independently of each other. Each state-machine contained the four validated states IC, FF, HO, TO as well as MS (Figure 2, panel E). Flags were used to ensure robustness of the algorithm. A flag can be considered a pre-event, an event that has to be detected to enable detection of the next stance event (orange dots in Figure 2). When a flag is raised, the state-machine does not change, and this only occurs upon detection of the respective stance-event, which, in turn, results in lowering the flag. An example in pseudo-code is shown in Algorithm 1, where the flag corresponds to a minimum and the event to a maximum.

In the update layer, a low-pass filtered (LPF) version of the corresponding signal was used to increase robustness of the detection and thus avoid false positives due to noise in the signal at the cost of a systematic delay. The update layer first detects the event using the filtered signal and subsequently uses the event detected in the filtered signal as a starting point to look for the true real-time (RT) event, thus resulting in thresholds for the filtered and raw signal based on these true events. At the start of each trial, generic thresholds based on data from two trials of two subjects were used. The thresholds were automatically updated every step in the update layer of the respective state-machine. Adaptive thresholds were updated based on the mean of the last five events, corrected for three times the standard deviation over these events and an offset. The experimentally set offset was included to reduce the influence of outliers, especially at gait onset. The number of standard deviations and the magnitude of the offset can be tuned manually, if deemed necessary. The example event included in Algorithm 2 corresponds to a maximum. The update layer ensures the adaptability of the algorithm to environmental changes or changes in the gait pattern. This structure was based on the three-layered algorithm presented in [6,21].

**Algorithm 1:** Real-time detection layer: Example of detecting event as a maximum
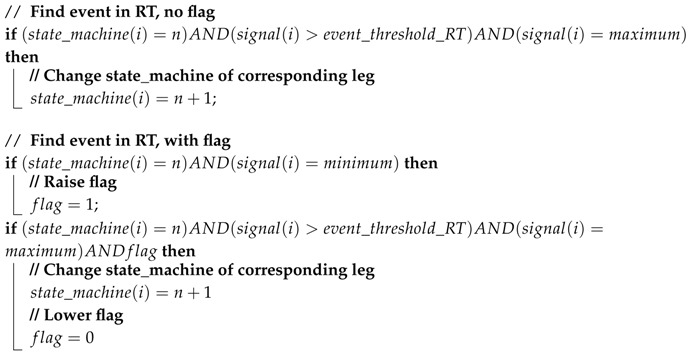


**Algorithm 2:** Update layer: Example event corresponding to a maximum
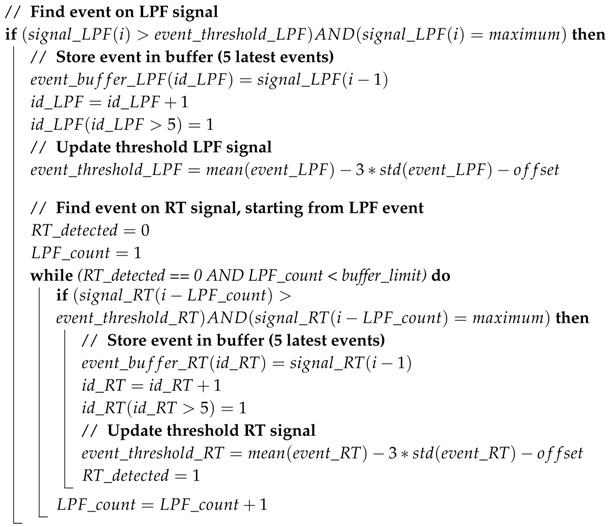


The following modifications with respect to the structure used in [21] were implemented.

The calibration layer was removed and replaced by generic thresholds. This enabled event detection from onset of walking, a requirement when embedded in NR control.

The update and real-time detection layer were decoupled, in order to make the algorithms computationally more efficient and to enable the use of different sample frequencies for the update layer and the detection layer.

To ensure correct identification of the end of the stance phase in the real-time detection, a secondary definition of TO was implemented. This secondary TO detection was only activated in case the initial TO event would go undetected. TO was then assumed to have occurred at the zero-cross of the shank angular velocity.

Finally, the global state-machine was replaced with an independent state-machine for each leg. The thresholds and adaptive policy were set conservatively since we prioritized precision over timing. Lower thresholds might result in a smaller delay but are also more prone to measuring noise and to result in false event detections.

The three algorithms: RTGSD-min, RTGSD-B6 and RTGSD-G6 are described below in more detail. Specific data required by each of the algorithms is extracted from a biomechanical model scaled to each subject (Figure 1 and Figure 2). RTGSD-min only requires a minimal data set consisting of sagittal plane shank angular velocity data and ankle angular data. It is therefore referred to as RTGSD-min and is particularly suited for AFO. RTGSD-B6 and RTGSD-G6 are geared towards a bilateral six degrees of freedom NP/NR, as indicated by the suffix 6 in their name. RTGSD-B6 is based on the event definitions as proposed by Botzel et al. [7], in particular the TO definition. RTGSD-G6 is an ambulatory implementation of the algorithm presented by Ghoussayni et al. [4]. All methods make use of sensors already needed for control of NP/NR/AFO. No additional sensors are needed, which means that cost and reliability are not affected.

### 2.4. RTGSD-Min

This algorithm was based on the stance events of [10] and the algorithm presented in [6]. A preliminary version of this algorithm was presented in [21], where data was obtained from potentiometers embedded in the exoskeleton. RTGSD-min only requires sagittal plane shank angular velocity data and ankle angular data. It is therefore particularly suited for AFO. This data can either be obtained through inertial sensors attached to the shank and foot (e.g., drop foot NP), an inertial sensor attached to the shank and an encoder providing ankle angle data (e.g., AFO [3,10]), or joint angle data from the ankle-knee-hip of each leg (e.g., gait exoskeleton [21]). In [21], no independent reference was available to validate the detected events.

IC was defined as the maximum shank angle (dashed line) with respect to the vertical. In agreement with [6], MS was defined as the maximum shank angular velocity (solid line) and TO as the minimum shank angular velocity. FF and HO were defined as, respectively, the first minimum and maximum of the ankle angle (dotted line) post ipsilateral IC (piIC), as in [21] (Figure 2C). Thresholds were attached to, and updated for, the detection of MS, IC and TO.

### 2.5. RTGSD-B6

RTGSD-B6 was developed according to the event definitions suggested by Botzel et al. [7]. IC was defined as the minimum of the shank angular velocity, MS as the maximum of the shank angular velocity (solid line). TO was defined as the midpoint between the trough and the zero-crossing of the shank angular velocity [7] (Figure 2B). The height of this threshold is dependent on walking speed [7]. The data provided in [7] was therefore spline fitted and interpolated to obtain the corresponding values (A) applicable to our data. The minimum of the shank angular velocity was used as a TO-flag after which the true TO event could occur, and its amplitude (M1) was used to update the threshold (M1*A) for the TO-detection of that gait cycle (Figure 2B). A similar flag was also used to enable HO detection and raised upon detecting the minimum in foot acceleration post-FF (dotted line). HO was defined as the minimum of the shank vertical position following FF (dashed line). The shank position was derived from the biomechanical model, rather than using marker position because the latter is not available outside of the lab. Botzel et al. did not propose a definition for FF, and the minimum in foot acceleration following IC was therefore used since pilot data revealed a high correlation between this signal and FF (Figure 2B). Thresholds for IC, FF, MS, and TO-flag were updated.

### 2.6. RTGSD-G6

The third algorithm (RTGSD-G6) is an ambulatory implementation of the algorithm presented by Ghoussayni et al. [4]. The original algorithm makes use of the sagittal plane velocities of the toe and heel markers. Marker data is not available outside of the lab but is assumed to represent the motion of the segments to which they are attached. We therefore computed the velocity of the scaled model every time step and extracted the velocity of the calcaneus and toe segments.

IC and HO were detected based on the vertical velocity of the calcaneus (dashed line). The vertical velocity of the toes (dotted line) was used to identify FF and TO. Only the vertical velocity component was taken into account, with the threshold levels fixed to 50 mm/s, based on the information in [4]. The magnitude of the segment vertical velocities was often below 100 mm/s; therefore, the threshold used in [4] for shod conditions was used (Figure 2A). Based on pilot results, the definition of IC was changed, with respect to [4], to correspond to the minimum in vertical velocity of the calcaneus. To make this IC detection robust, an IC-flag was raised by the zero crossing (ZC) of calcaneus vertical velocity from positive to negative post-MS. MS is the only event for which the threshold was updated.

## 3. Results and Discussion

### 3.1. Results

**Figure 3:**
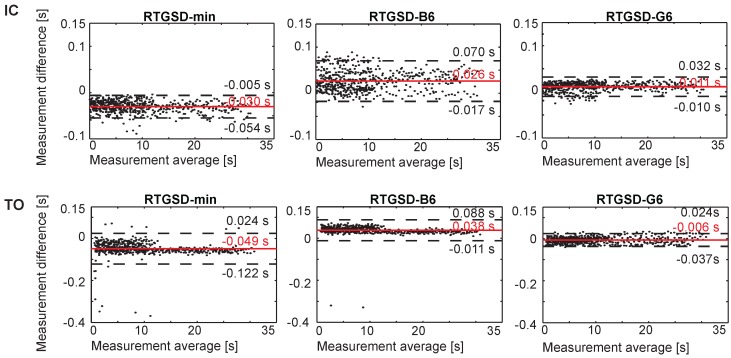
Bland–Altman plots for IC and TO of walking at 3, 4, 5 km/h on both left and right legs of seven healthy subjects. Positive times reflect delays of the presented method with respect to the reference. A solid grey line indicates mean error, and the confidence interval (mean ± 1.96 SD) is represented by discontinuous black lines.

**Figure 4:**
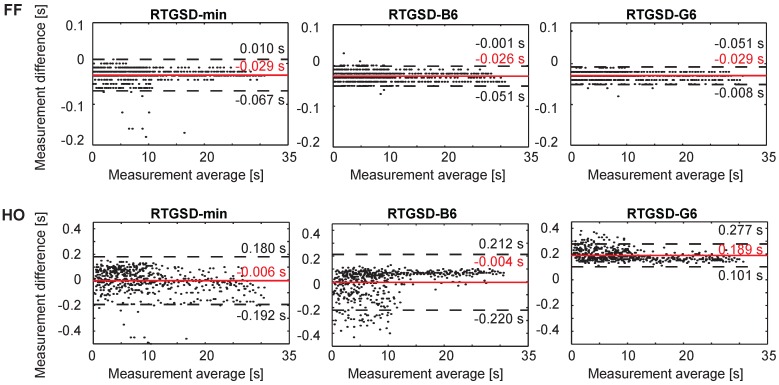
Bland–Altman plots for IC and TO of walking at 3, 4, 5 km/h on both left and right legs of seven healthy subjects. Positive times reflect delays of the presented method with respect to the reference. A solid grey line indicates mean error, and the confidence interval (mean ± 1.96 SD) is represented by discontinuous black lines.

A total of 558 steps were analysed by each algorithm and compared against reference data based on a Vicon motion capture system (Oxford Metrcis Group, Oxford, UK) and force plates, the ground truths for kinematics and gait events. All algorithms achieved a precision score equal to one for all events, meaning that no false positives occurred. False negatives were only found for RTGSD-B6, corresponding to three HO events. The detection delays for each algorithm are represented below in Bland–Altman plots for IC and TO (Figure 3), and FF and HO (Figure 4). In each panel, the difference between the reference and the respective algorithm was plotted against their average. Positive times reflected a delay of the algorithm under consideration with respect to the reference. Results from left and right state machines were similar and were therefore combined. Discontinuous lines mark the 95% confidence interval. The mean delays (and standard deviations), in ms, are summarized in Table 2.

RTGSD-G6 had the smallest mean delay and variability for IC and TO detection (Figure 3). In general, all three algorithms performed similarly on these events. The results for RTGSD-B6 did not confirm that the TO definition applied in this algorithm results in improved mean delay and/or variability with respect to the other algorithms. FF was detected equally well by all algorithms, with mean delays around those observed for IC and TO detection (Figure 4). However, HO detection resulted either in large mean delay (RTGSD-G6) or large variability (RTGSD-min and RTGSD-B6).

### 3.2. Discussion

Three novel real-time algorithms were presented, detecting four separate gait events (IC, FF, HO and TO) relevant for controlling an NP/NR. The presented algorithms are based on kinematic data available in most NP/NR, or in standard gait monitoring and analysis. The novelty of the presented algorithms lies in using kinematic data and coupling it to a biomechanical model. This enables to online extract features and signals commonly used in the vast body of literature on gait event detection online that are otherwise not available online. In this paper, this innovative approach was coupled to a threshold-based structure where the state-machine to detect the events and the updating of the thresholds used in the detection are performed in parallel. All presented algorithms detected IC, FF and TO well within the established limits of 61 ms (125 ms), allowing time for additional delays stemming from the control and hardware, as can be seen from Figure 3 and Figure 4 and Table 2.

The performance of the presented algorithms also matches the current state of the art for real-time IC and TO detection. Chia et al. reported delays of, respectively, 13 ms and 10 ms for IC and TO [6]. The delays reported by Pappas for healthy subjects walking on a treadmill are 70 ms for IC, 70 ms for FF, 40 ms for HO, and 35 ms for TO [12]. The algorithm presented by Pappas et al. is one of the few wearable solutions to detect IC, FF, HO and TO in real time, and is often used as a benchmark in literature. RTGSD-min and RTGSD-B6 outperform the algorithm of Pappas on a very similar dataset, despite not relying on footswitch data. RTGSD-G6 produced better results for IC, FF and TO but not for HO.

Comparing results of different algorithms across studies is often difficult since other references may have been used to design and/or validate the respective algorithms. These differences are likely to lead to small differences in event detection between the respective algorithms, and also compared to the reference at hand. In RTGSD-min, the definitions as used in [6] were implemented, yet higher mean delays were observed. In [6], IC definition was reported to be tailored to the reference used, a GaitRite system. It is possible that the definition provided by [6] deviates slightly from the force plate event used in this study. In the updating of the thresholds, we favoured precision over timing, although taking a smaller safety margin on the thresholds could have resulted in reduced delays. In RTGSD-B6, IC was defined, in agreement with the consensus in literature, as the minimum of shank angular velocity; delays similar to those in RTGSD-min were obtained. In this study, we used force platform data to obtain the reference for IC and TO. The definition of IC and TO in RTGSD-G6 was adapted from [4] where force platform data was also used as a reference, producing results that outperformed the current state of the art. The mismatch between event definitions between the respective algorithms and the reference is likely responsible for the observed small differences in IC, FF, and TO detection between algorithms. The higher delay in TO for RTGSD-min and RTGSD-B6 with respect to the results of [6] was also in part due to few outliers. In these cases, the respective TO definition was not engaged, due to the threshold not being fulfilled, and TO was not detected until the shank angular velocity became positive. The latter was implemented as a secondary TO definition to compensate for the decoupling between the update and real-time detection layer. In [6], there was a stronger coupling between both layers, where corrections can occur from the update to the detection layer. To reduce the computational load, this coupling was removed in the structure used in this study. If sufficient computational power is available, restoring this coupling could thus result in improved TO detection delays. The new TO definition proposed in [7] was implemented in RTGSD-B6 but did not appear to outperform the two other algorithms (Table 2).

Threshold-based HO detection appears problematic. Kotiadis et al. reported detection delays of, respectively, 5 ms and 100 ms for HO in their two best performing algorithms [11]. However, this data was only of one subject and the algorithm with the lowest delay on HO had a mean delay of 70 ms on IC. RTGSD-min and RTGSD-B6 had small mean delays for HO but displayed a very large variability. The definition of HO recently proposed in [7] and implemented in RTGSD-B6 resulted in the lowest mean delays, but, like all other methods, suffered from large variability and also had three false negatives. The variability in RTGSD-G6 was lower, but the mean delay of −189 ms fell outside of the defined limits. Given that the other events can be detected with much smaller delays and that the order of gait events is inherently sequential, future efforts should consider coupling a probabilistic method or a machine learning method to threshold-based algorithms to decrease the variability and mean delay of HO detection.

The results obtained in this study were obtained offline, based on joint kinematics obtained from a Kalman Smoother algorithm. In real-time applications, it is reasonable to expect slightly higher delays, depending on the quality of the real-time joint kinematics and filtering performed. However, given the characteristics of joint kinematics, these additional delays are even in the worst-case scenario not expected to exceed 10 ms. Future studies should validate the presented approach and/or algorithms on NR/NP, and over a wider range of activities.

## 4. Conclusions

A novel structure was presented where joint kinematics are coupled to a biomechanical model, scaled to the subject, to detect gait events. The scaled model allows extracting features otherwise unavailable outside of the laboratory, or unavailable from kinematics alone. Three algorithms were presented based on this structure for the detection of IC, FF, HO and TO in NR/NP. The algorithms were threshold based and a computationally efficient structure was applied to facilitate embedding the algorithms on portable devices. Since no sensors other than those already present for the control of the NP/NR/AFO are required, cost and reliability of the NP/NR/AFO remain unchanged. The very low delays observed and 100% precision scores suggest that these algorithms can be used in combination with real-time NP/NR control algorithms. However, care should be taken when using the HO event since higher variability was observed for this event. Our results suggest that threshold based methods might not be the most suitable approach to detect HO online. Future studies should therefore look into enhancing threshold based algorithms with probabilistic methods to reduce HO variability. The presented methods can also be used on gait monitoring, screening and follow-up of pathologies. In our future work, we will apply these detection methods in the control of an exoskeleton.

## Figures and Tables

**Table 1:** Summary of stance events as defined by each of the three algorithms.

Event	RTGSD-Min	RTGSD-B6	RTGSD-G6
IC	max. tibia angle	min. tibia angular vel.	calcaneus vertical vel. > −50 mm/s
FF	1st min. ankle angle, piIC	min. foot angular acc.	toes vertical vel. > −50 mm/s
HO	1st max. ankle angle, piIC	min. tibia vertical position	calcaneus vertical vel. > 50 mm/s
TO	min. tibia angular vel.	midpoint min. and ZC tibia angular vel.	toes vertical vel. > 50 mm/s

**Table 2:** Mean detection delays in ms (±standard deviation) of the developed algorithms for each of the four stance phases evaluated. The results achieved by the real-time algorithm with lowest reported delays [6], and the best performing real-time algorithm detecting all events (with use of FSR) [12], have been included for comparison.

Algorithm	IC (ms)	FF (ms)	HO (ms)	TO (ms)
RTGSD-min	−30.45 (12.45)	−28.54(19.65)	5.83 (95.15)	−49.08(37.14)
RTGSD-B6	26.53 (22.43)	26.21 (12.48)	3.63 (110.37)	38.52 (25.35)
RTGSD-G6	11.10 (10.72)	−29.33(11.00)	189.39 (44.99)	6.40 (15.49)
Chia [6]	13	N.A.	N.A.	10
Pappas [12]	70	70	40	35

## Abbreviations

The following abbreviations are used in this manuscript:

AFO	Ankle-Foot Orthoses
FF	Foot Flat
FSR	Force Sensitive Resistor
GRF	Ground Reaction Forces
HO	Heel Off
IC	Initial Contact
LPF	Low-Pass Filtered
MS	Mid Swing
NP	NeuroProsthetics
NR	NeuroRobotics
piIC	post ipsilateral IC
RT	Real-Time
RTGSD	Real-Time Gait and Stance Detection
TO	Toe Off
ZC	Zero-Crossing
